# Surprising Solid-State ESIPT Emission from Apparently Ordinary Salicyliden Glycinates Schiff Bases

**DOI:** 10.3390/ijms232314955

**Published:** 2022-11-29

**Authors:** Mateusz M. Tomczyk, Łukasz Przypis, Tomohiro Shiraki, Joachim Kusz, Maria Książek, Dawid Janas, Nikodem Kuźnik

**Affiliations:** 1 Faculty of Chemistry, Silesian University of Technology, M. Strzody 9, 44-100 Gliwice, Poland; 2 Saule Research Institute, Wroclaw Technology Park, 11 Dunska Street, Sigma Building, 54-130 Wroclaw, Poland; 3 Department of Applied Chemistry, Graduate School of Engineering, Kyushu University, 744 Motooka, Nishi-ku, Fukuoka 819-0395, Japan; 4 International Institute for Carbon-Neutral Energy Research (WPI-I2CNER), Kyushu University, 744 Motooka, Nishi-ku, Fukuoka 819-0395, Japan; 5 Institute of Physics, University of Silesia, 75 Pułku Piechoty 1, 41-500 Chorzów, Poland

**Keywords:** Excited State Intramolecular Photon Transfer, ESIPT, salicyliden glycinates, Schiff base, keto-enol tautomerism, alkali metals coordination, solid state fluorescence

## Abstract

Excited-State Intramolecular Photon Transfer (ESIPT) is known for the geometry-related phenolic and imine groups. The Schiff bases formed upon condensation of salicyl aldehyde and glycine led to the formation of ESIPT models. A series of alkali metal salicyliden glycinates were analyzed by X-ray diffraction of their monocrystals and spectroscopy measurements. The X-ray analysis revealed varied hydration levels between the salts. They adapted trans geometry on the imine groups and mostly anticlinal conformation with the neighboring atoms, which is different from the other structurally-related compounds in literature. Fluorescence of these compounds was found for the crystalline forms only. Protonation of the imine nitrogen atom and further proton distribution was consistent with the ESIPT theory, which also explained the observed fluorescence with the highest Stokes shift of 10,181 cm^−1^ and 10.1% of fluorescence quantum yield for the sodium salt.

## 1. Introduction

**Scheme 1:**
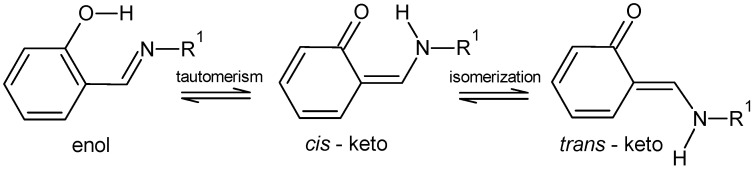
Common ESIPT models.

Excited-State Intramolecular Photon Transfer (**ESIPT**) materials have received considerable attention in recent years due to unique features such as large Stokes shift and broad emissions, which might be useful for producing cheap white light-emitting diodes and devices for lightning or illumination [1]. The fundament of the process is the formation of an intramolecular hydrogen bond between the donor and acceptor fragment of a molecule that exhibits keto-enol tautomerism (Scheme 1). It has been identified that the isomerization between *cis*- and *trans*-keto forms is related to the photo- or thermochromism of these compounds [2,3,4,5,6].

Since the discovery of the ESIPT in the salicylic acid molecule by Weller et al. in 1955 [7], a range of ESIPT fluorophores were discovered and designed, such as 2-(2′-hydroxyphenyl)benzimidazole, [8] derivatives of quinoline, [9] thiodiazoles [10] or salicyliden aniline [11] with high emission efficiencies in solution but weak in the solid state, although the latter is more advantageous for optoelectronic applications [12]. In order to introduce, improve or modify ESIPT emissions in the solid state, many strategies were developed utilizing, e.g., aggregation-induced emission (AIE), aggregation-induced enhanced emission (AIEE), molecular packing modification, or molecular design of chromophores with polymorphic properties [12,13,14]. Recently, we reported the synthesis of simple salicyliden imine of potassium glycinates (GlySalK: GlySalK-a and GlySal-h) [15]. The compound exhibited polymorphism, and the color of crystals changed significantly from red to yellow upon hydration. During further investigation of the compound, we also discovered it is fluorescent in the solid state, most likely due to the ESIPT process.

This article investigates the origin and nature of the features described above. Moreover, the fluorescence source is elucidated and explained based on the ESIPT process, occurring only in the solid state due to AIE. Lastly, the effect of the alkali metal cation on the emission properties was considered.

## 2. Results

**Scheme 2:**
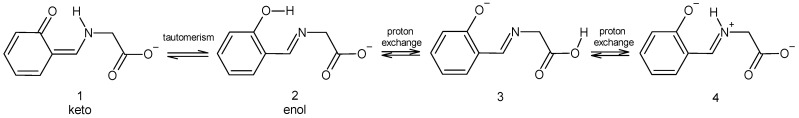
Salicyliden imine of glycinate GlySlaX and its tautomerism and proton exchange between oxygen atoms.

The **GlySalX** compounds are formally imines of salicylic aldehyde and lithium (**GlySalLi**), sodium (**GlySalNa**), or potassium (**GlySalK**) glycinate (Scheme 2) crystalizing easily from methanol solution, and soluble only in alcohols, while prolonged water treatment leads to their hydrolysis. Methanol is the solvent of choice for this particular group of compounds, as the water concentration of ethanol is too high, while in 1-propanol the solubility significantly decreases.

Because the compound has a carboxylic group, it can be easily converted to alkali metal salts. Interestingly, the color of those salts changes with the cation. The imines exhibit a bathochromic change of color in the solid state from light lime **GlySalLi-a** to lime **GlySalNa-h**), and then to orange for anhydrous **GlySalK-a**. It has to be mentioned that the color of those salts turns yellow upon hydration (**GlySalX-h**). The difference in the color of the solids induced by the change of the metal center indicates a strong modification of molecular arrangement in the solid state.

### 2.1. Crystal Structure

**Figure 1:**
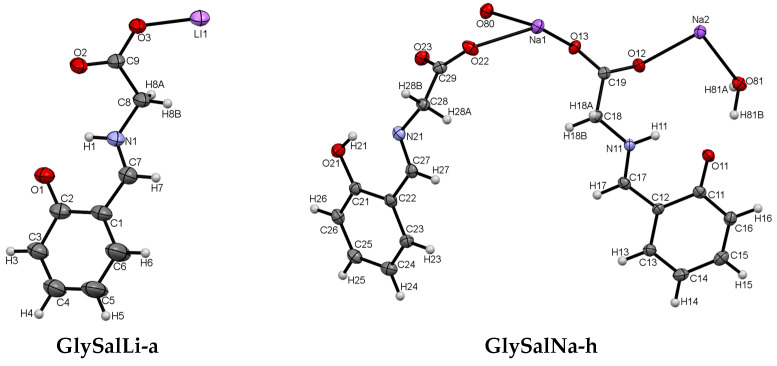
X-ray crystal structures of the asymmetric units of **GlySalLi-a** and **GlySalN-h** showing the numbering schemes. Displacement ellipsoids are shown at 50% probability level.

To explore this hypothesis further, X-ray single crystal structures of compounds **GlySalLi-a** and **GlySalNa-h** were obtained—Figure 1. The most important crystallographic information for both compounds is gathered in Table 1.

**Figure 2:**
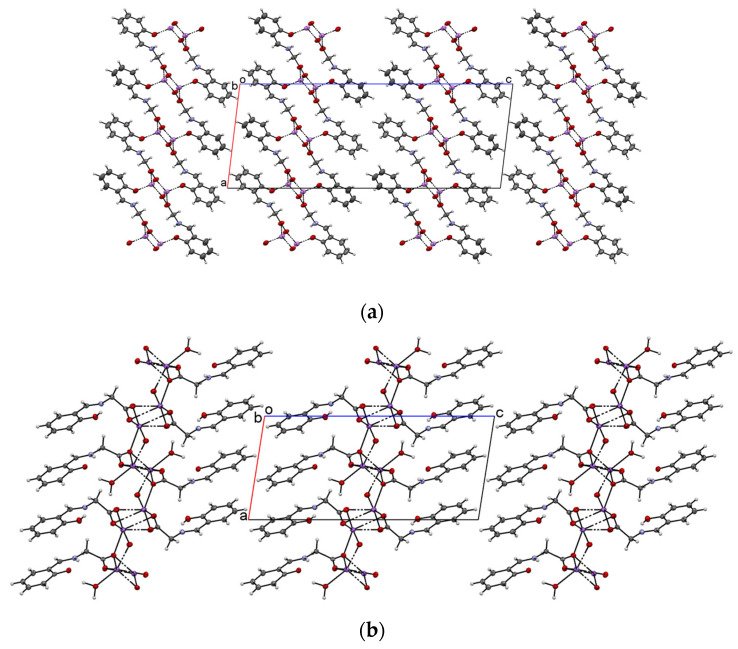
Crystal packing for (**a**) **GlySalLi-a** and (**b**) **GlySalNa-h**.

**GlySalLi-a** crystalizes as anhydride in monoclinic unit cell *I*2/a. Moreover, **GlySalNa-h** forms monohydrated crystals in a monoclinic unit cell *P*2_1_ with two different molecules in its asymmetric part. The crystal packing showing the propagation of the polymeric structure is shown in Figure 2. In both cases, the molecules create layers, which are parallel to the [010] direction.

In **GlySalLi-a,** the phenolic oxygen atoms form a relatively short bond with the aromatic ring of 1.282(5) Å and creates an ionic interaction with Li cations. Therefore, the “ESIPT proton” in the crystal structure is located on the imine nitrogen atom N1 (forms **1** and **4** according to Scheme 2). In the case of **GlySalNa-h**, one of the molecules forms a typical, protonated phenol (H-O21), imine (N21) with deprotonated carboxylic groups (form **2**—Scheme 2). However, the other molecule, similarly to the **GlySalLi-a** has a protonated imine (N11) with deprotonated phenolic oxygen atom (O11) with a shorth C-O bond 1.289(4) Å (forms **1** and **4**), but forms a hydrogen bond with a water molecule.

The analysis has also shed new light onto a variety of conformation of GlySalX along C7-N1-C8-C9 imine bond depending counterion and hydration—Table 2. As we reported before, GlySalK-a adapts antiperiplanar while GlySalK-h is in anticlinal conformation (torsion 172.38(11) and 124.4(4), respectively) [15].

All the GlySal ligands presented here bear *trans* geometry on the imine moiety. However, a comparison of the **GlySal** ligand in the deprotonated with different alkali metals (Li-K) reveals the influence of the cations on the structure. **GlySalLi** and both forms of **GlySalK** are characteristic of imine protonation (forms **1** and **4**). At the same time, the sodium counterpart **GlySalNa** is the only structure found in the series with a typical imine moiety (structure 2). However, the position of the proton does not translate to the conformation along the CN skeleton (C7-N1-C8-C9), which is varied, mostly anticlinal, and unique antiperiplanar for **GlySalK-a**. Moreover, as it was reported before, the torsions are the largest since copper or nickel complexes coordinated with similar skeleton express only smaller leaning of the bulky groups out from coplanarity [15]. Additionally, the coordination of the GlySal ligand in the Li-K series presents characteristic coordination numbers: 4 for **GlySalLi**, 6 for **GlySalNa**, and 7 for both **GlySalK** forms.

In both structures (i.e., **GlySalLi-a** and **GlySalNa-h**) there are present intra- and intermolecular hydrogen bonds. Table 3 contains geometric parameters of the hydrogen bonds present in both structures.

**Figure 3:**
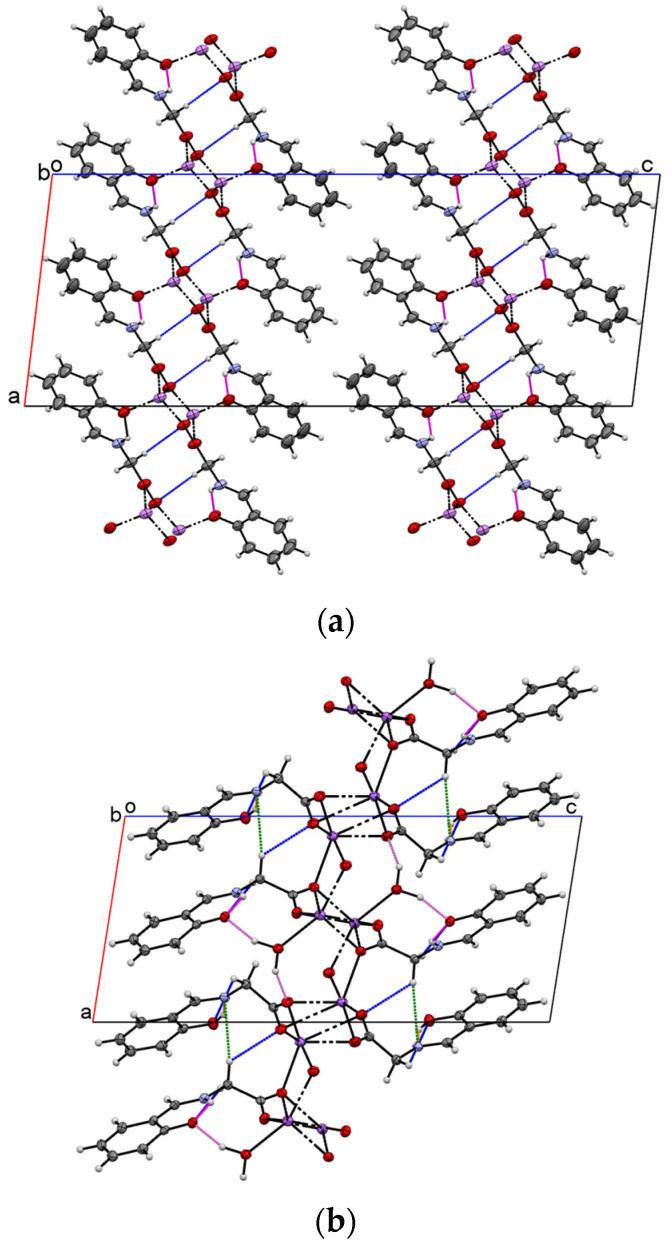
Hydrogen bonds for (**a**) **GlySalLi-a** and (**b**) **GlySalNa-h**. C-H···O marked by blue, N-H···O—by magenta, C-H···N—by green, O-H···N—by orange, O-H···O—by light violet, dashed lines.

The structure **GlySalLi-a** is stabilized by intramolecular hydrogen bond between N1 atom and O1 atom (Figure 3a). There is also an observed intermolecular hydrogen bond between the C18 atom and O3 atom (Figure 3a). According to the characteristic of the hydrogen bonds proposed by Desiraju and Steiner [16], the interaction of the type N-H···O can be classified as a strong, conventional hydrogen bond, while the interaction of the type C-H···O is a weak, non-conventional hydrogen bond.

The **GlySalNa-h** structure is also stabilized by an intramolecular hydrogen bond, namely between the N11 atom and O11 atom and between the O21 atom and N21 atom (Figure 3b). Taking into account the geometric parameters of these interactions (Table 3), they can be classified as strong, conventional hydrogen bonds. Moreover, there occurs several intermolecular interactions in the crystal structure of **GlySalNa-h**, as for example C18-H18A···N21(*x*, *y*−1, *z*), C18-H18A···O23 or C18-H1B···O11(*x*, *y* + 1, *z*) (Figure 3b). All of them belong to the weak, non-conventional hydrogen bonds (Table 3). Water molecules, which are present in the structure, create strong, conventional hydrogen bonds (i.e., O81-H81A···O22(*x*−1, *y*−1, *z*) and O81-H81B···O11).

### 2.2. Absorption and Emission Properties

**Figure 4:**
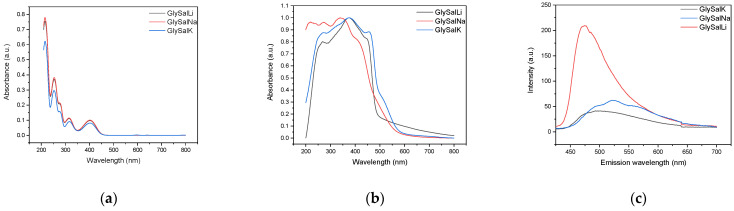
Optical absorption spectra of GlySalX in (**a**) methanol; and (**b**) solid form. (**c**) Corresponding emission in solid form.

Solution spectra in methanol of **GlySalX** are almost indistinguishable, with only minor differences between the position of absorption bands and the molar absorption coefficient of n-p* or p-p* transitions (Figure 4).

Furthermore, none of the compounds show detectable fluorescence properties in the solution, most likely due to the hydrogen interactions with solvent molecules’ freedom of Z/E isomerization, which dissipates the ESIPT process. [1] All compounds exhibit three overlapping absorption bands in the solid state, with the medium-energy absorption band associated with n-p* transitions having the highest absorption coefficient. In methanol, four well-resolved absorption bands appear, with only one n-π* transition in the visible range. The lowest-energy absorption band in the solid-state associated with p-π* transitions from the metal center to phenol or carboxy group is red-shifted for **GlySalK** compared with **GlySalLi**. This result indicates that the metal center is responsible for its position. Moreover, it is invisible in the solution, indicating the metal center’s separation from the anion fragment through solvent molecules.

Among **GlySal** compounds, **GlySalLi** exhibits the most blue-shifted fluorescence with an emission maximum at 470 nm, while **GlySalK** and **GlySalNa** emission maxima are at 500 and 520 nm, respectively, Table 4. To compare the influence of the metal ions on the emission, we calculated Stokes shifts which resemble the transition energy. Only for **GlySalNa** a substantial value of 10,181 cm^−1^ was observed, while for **GlySalLi** and **GlySalK** shifts were smaller although still considerably high [17]. PL QY values are relatively low despite the large Stokes shift, most likely due to the self-absorption as the fluorescence spectra of **GlySal** compounds partially overlap with the lowest energy absorption band in the solid state.

In general, these values support the observation that fluorescence is due to the ESIPT process, which induces large changes in the molecule’s dipole moment [1]. In **GlySalNa** crystal structure, the phenolic oxygen atom is not involved in the coordination of the metal center. Thus, it has a relatively higher negative charge than **GlySalLi** and **GlySalK**. Based on the crystal structures, it could be hypothesized that in **GlySalK** and **GlySalLi** the keto-enamine is responsible for the absorption process. In contrast, for **GlySalNa** both tautomers are involved in absorption and emission processes. Such a situation is not often found in ESIPT molecules as typically the enol-form is the ground state responsible for absorption while keto-form is for ESIPT emission.

**Figure 5:**
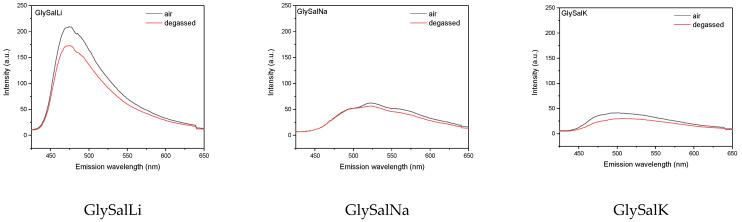
Emission spectra in the solid state of GlySalX in air and after degassing (λ_Ex_ = 337 nm).

**Figure 6:**
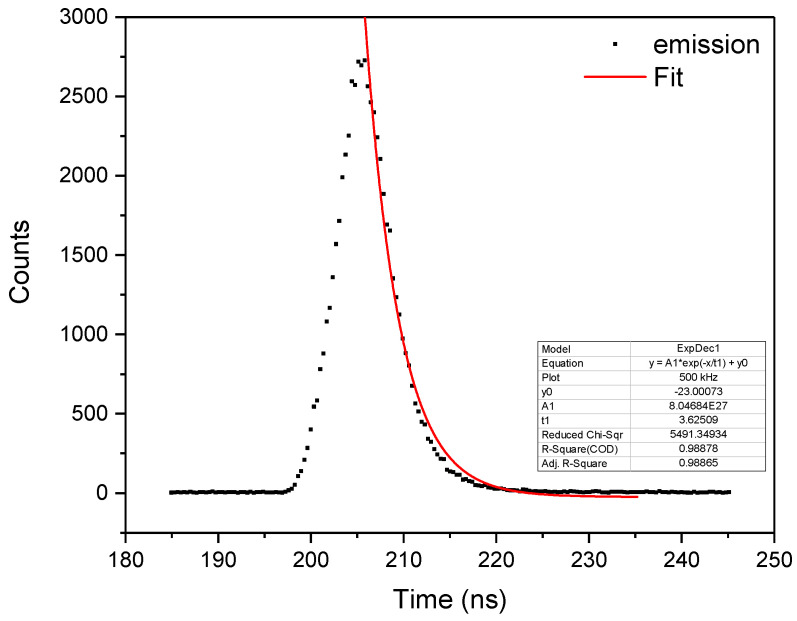
Time domain intensity decay of GlySalLi. The instrument response function (**black squares**) and exponential decay fit (**red line**).

**Figure 7:**
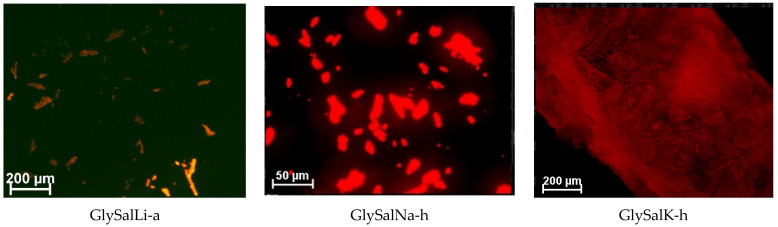
Confocal microscope images of GlySalX crystals ex green laser, blue and green light filtered off.

The characteristic property of ESIPT fluorescence is that it has a short lifetime in the range of nanoseconds. Thus, we performed time-correlated single photon counting. To verify whether a triplet state is involved and potentially limiting fluorescence through non-radiative processes, we investigated the emission properties of **GlySalX** compounds in air and under vacuum—Figure 5. For all **GlySalX** compounds, there was no noticeable increase in the fluorescence intensity upon degassing, which indicates that oxygen atom is not involved in the formation of triplet states. In fact, there is an evident luminescence decrease which we associate with the physical process of tightening solid particles under vacuum during the measurement. Lastly, we measured the fluorescence lifetime, which was 3.5 ns for GlySalLi, in the range of the fluorescence lifetimes expected from ESIPT fluorophores—Figure 6 [1]. Concomitantly, solid-state fluorescence of GlySalX crystals has been recorded, which visualized the impact of alkali metal cation on the emission properties of studied materials—Figure 7.

## 3. Materials and Methods

### 3.1. Synthesis of Salicyl-Glycine Schiff Bases Salts GlySalX (GlySalLi or GlySalNa)

Glycine (5.0 g, 67 mmol) and appropriate hydroxide XOH (67 mmol) were dissolved in methanol (50 mL). A solution of salicylaldehyde (8.1 g; 66 mmol) in methanol (50 mL) was added. The color of the solution rapidly turned yellow with the formation of a colored precipitate. The mixture was stirred for 1 h at room temperature. The resulting solid product was filtered and air-dried. Yield 74%, (49 mmol) m.p. = 219–221 °C.

### 3.2. Solid State and Solution Fluorescence Measurements

Excitation and emission spectra in the visible region were recorded using a Camlin spectrometer (Lisburn, Northern Ireland, UK) equipped with a Xe-lamp as a light source, monochromator, and Si-based detector. All spectra were normalized to upcoming light intensity. The PL QY was measured using an integrating sphere.

### 3.3. UV-Vis Absorption Spectroscopy

UV-vis region spectra were recorded using Ocean Optics QE spectrometer (Dunedin, Florida, USA) multi-channel silicon-based array detector with 1044 pixels. The final spectrum is an average of 10 spectra recorded with 100 msec integration time, with boxcar smoothing equal to 5 with dark spectrum correction.

### 3.4. NMR Spectroscopy

The ^1^H and ^13^C NMR spectra were recorded at 298 K on an Agilent NMR Magnet−400 MHz 400 MHz using tetramethylsilane as an internal standard or solvent residual protons. Chemical shifts CD_3_OD (δ = 3.34 ppm for ^1^H NMR and δ = 49.86 ppm for ^13^C NMR). Coupling constants (*J*) are reported in hertz (Hz). The multiplicity of the signals is given as s (singlet), d (doublet), t (triplet), and q (quarter).

For **GlySalNa** ^1^H NMR: 8.32 (s, 1H, CAr-CH=N), 7.30 (d, *J* = 7.6 Hz, 1H o-CAr-H), 7.27 (dd, *J* = 8.2, 7.4 Hz, 1H, p-CAr-H), 6.78 (dd, *J* = 8.2, 0.8 Hz, 1H, m-CAr-H-CAr-OH), 6.70 (ddd, *J* = 7.6, 7.4, 0.8 Hz, 1H, m-CAr-H-CAr-H), 4.21 (m, 2H, =N-CH2).

For ^13^C NMR: 176.7 (COO), 168,8 (CH=N), 161.9 (C_Ar_-OH), 135.6 (p-C_Ar_-H), 134.4 (o-C_Ar_-H), 120.5 (m-C_Ar_-H), 118.8 (C_Ar_-CH = N), 118.8 (C_Ar_-H-CAr-OH), 61.9 (=N-CH_2_). MS for C_9_H_9_NO_3_ [M, GlySal]+: calculated = 180.1; found: 180.2.

### 3.5. X-Ray Crystallography

The data for **GlySalLi-a** and **GlySalNa-h** were collected using SuperNova diffractometer (Rigaku Oxford Diffraction, Oxford, UK) equipped with Atlas CCD detector and using Cu Kα radiation at 100 K. Unit cell parameters were determined and refined using CrysAlis^Pro^ program [18]. Additionally, the integration of the collected data was performed with the same program. Both structures were solved using direct methods with SHELXS-2013 program and then refined using SHELXL-2019/2 program [19]. Nonhydrogen atoms were refined with anisotropic displacement parameters. The hydrogen atoms were fixed at calculated distances and allowed to ride on the parent atoms using suitable constraints in SHELXL [19].

The crystal structures CIF files for GlySalLi-a and GlySalNa-h have been deposited in a Crystallography Open Database (crystallography.net) under No. 3000421 and 3000422, respectively. The crystal structures have been also deposited at the Cambridge Crystallographic Data Centre. CCDC-2220609 and 2220610 contain the supplementary crystallographic data for this paper. The data can be obtained free of charge via Cambridge Crystallographic Data Centre: www.ccdc.cam.ac.uk/structures (accessed 26 November 2022).

### 3.6. Confocal Microscopy

Confocal microscope images were acquired using LSM 510 confocal microscope (Carl Zeiss, Jena, Germany).

## 4. Summary and Conclusions

New Schiff base models were synthesized from salicyl aldehyde and glycine salts of alkali metals Li, Na, and K. They formed crystals of different water content. In their crystal structures, protons were distributed among N and O heteroatoms. The most acidic carboxylic group was not protonated. However, only one of two symmetrically independent molecules in the sodium salt crystals bore the proton on the phenolic oxygen atom. The imine moiety’s conformation was unique compared to previously reported compounds with structurally related motifs. The proton distribution and exchange were specific to Excited State Intramolecular Photon Transfer (ESIPT) which was observed as fluorescence in the solid state. While the fluorescence was muted in solutions, it reached the 10,181 cm^−1^ shift for the sodium salt in crystals, which was consistent with the ESIPT phenomenon. The contribution of triplet oxygen atom in the photochemical process could be neglected due to the negative fluorescence tests in degassed samples. Therefore, the solid-state fluorescence and the structural features of the salicyliden glycinates support the ESIPT theory. Additionally, the relation of the coordination number, imine geometry and hydrogen distribution in the crystalline structures’ alkali metals (Li-K) salicyliden glycinates on their spectroscopy characteristics was observed.

## Figures and Tables

**Table 1:** Crystallographic information concerning **GlySalLi-a** and **GlySalNa-h** structures.

	GlySalLi-a	GlySalNa-h
Empirical formula	C_9_H_8_LiNO_3_	C_18_H_18_N_2_Na_2_O_8_
Molecular weight	185.10	435.32
Temperature [K]	100	100
Crystal system	Monoclinic	Monoclinic
Space group	*I*2/a	*P*2_1_
Unit cell dimensions		
a [Å]	11.6482(9)	8.6872(2)
b [Å]	5.0003(4)	5.8744(1)
c [Å]	30.317(2)	19.0522(6)
β [°]	96.865(8)	98.906(2)
V [Å^3^]	1753.1(2)	960.55(4)
Z	8	2
Density (calculated) [Mg/m^3^]	1.403	1.509
Absorption coefficient [mm^−1^]	0.869	1.393
F(000)	768	452
Theta range for data collection	2.936 to 74.822	4.699 to 73.696
Index ranges	−14 ≤ *h* ≤ 10	−10 ≤ *h* ≤ 10
	−5 ≤ *k* ≤ 6	−7 ≤ *k* ≤ 6
	−37 ≤ *l* ≤ 36	−23 ≤ *l* ≤ 22
Reflections collected	4141	6598
Independent reflections	1685	3200
R(int)	0.0636	0.0258
Completeness to theta = 67.684° [%]	98.4	99.9
Data/restraints/parameters	1685/0/127	3200/0/273
Goodness-of-fit on F^2^	1.031	1.071
Final R indices [I > 2σ(I)]	R1 = 0.0714 wR2 = 0.1735	R1 = 0.0361 wR2 = 0.0967
R indices (all data)	R1 = 0.1245 wR2 = 0.2119	R1 = 0.0381 wR2 = 0.0986
Largest diff. peak and hole [eÅ^−3^]	0.289 and −0.328	0.480 and −0.310

**Table 2:** Torsion angles along C7-N1-C8-C9 bonds for GlySalX structures, where X—selected alkali metals. Anhydrous compounds are denoted with a, while hydrated with h.

Structure	Torsion Angle [°]	Conformation
**GlySalLi-a**	135.4(4)	*tilted* anticlinal
**GlySalNa-h**	118.3(3); 139.7(3)	anticlinal; *tilted* anticlinal
**GlySalK-a** *	172.38(11)	antiperiplanar
**GlySalK-h** *	124.4(4)	anticlinal

* published before [15].

**Table 3:** Hydrogen bonds for GlySalLi-a and GlySalNa-h.

Compound	D-H···A	d(D-H)	d(H···A)	d(D···A)	<(DHA)
**GlySalLi-a**	N1-H1···O1	0.88	1.87	2.574(4)	136.0
	C8-H8A···O3 (i)	0.99	2.52	3.468(5)	160.6
**GlySalNa-h**	N11-H11···O11	0.88	1.89	2.595(4)	135.9
	C18-H18A···O23	0.99	2.61	3.309(3)	127.3
	C18-H18A···N21 (ii)	0.99	2.67	3.651(3)	168.6
	C18-H18B···O11 (iii)	0.99	2.50	3.389(4)	149.1
	C28-H28A···O21 (ii)	0.99	2.71	3.351(4)	122.8
	O21-H21···N21	0.84	1.85	2.596(4)	147.2
	O81-H81A···O22 (iv)	1.04	1.68	2.659(3)	154.1
	O81-H81B···O11	1.05	1.73	2.714(3)	155.0

D—donor in a hydrogen bond, A—acceptor in a hydrogen bond, d—distance. Symmetry codes: (i) −*x* + 1/2, −*y* + 5/2, −*z* + 1/2; (ii) *x*, *y*−1, *z*; (iii) *x*, *y* + 1, *z*; (iv) *x*−1, *y*−1, *z*.

**Table 4:** Photophysical data of GlySalX in the solid state.

Compound	Absorption (nm)	Emission (nm)	Stokes Shift (cm^−1^)	PLQY (%)
**GlySalLi**	373	470	5533	12.1
**GlySalNa**	340	520	10,181	10.1
**GlySalK**	378	500	6455	8.1

## Data Availability

Data from this manuscript can be obtained from the corresponding author upon a reasonable request.
